# The Healthcare Professionals’ and Patient Advocates’ Perspectives on the Care for Children with Cancer in Europe—A Report from the ESCALIER Project

**DOI:** 10.3390/curroncol32020084

**Published:** 2025-01-31

**Authors:** Maria Otth, Marko Ocokoljic, Theodora Armenkova, Irina Ban, Samira Essiaf, Maximilian Hopfgartner, Lejla Kameric, Pamela R. Kearns, Georgia Kokkinou, Carmelo Rizzari, Carina Schneider, Katrin Scheinemann

**Affiliations:** 1 Division of Oncology-Hematology, Children’s Hospital of Eastern Switzerland, 9006 St. Gallen, Switzerland; 2 Department of Oncology, University Children’s Hospital Zurich, 8008 Zurich, Switzerland; 3 Faculty of Health Sciences and Medicine, University of Lucerne, 6002 Lucerne, Switzerland; 4 The European Society for Paediatric Oncology (SIOP Europe), 1200 Brussels, Belgium; 5 Childhood Cancer International-Europe (CCI Europe), 1080 Vienna, Austria; 6 Foundation Gold, 1836 Sofia, Bulgaria; 7 ZVONČICA Association of Children with Cancer and Other Rare Diseases, 11070 Belgrade, Serbia; 8 Heart for Kids with Cancer, 71000 Sarajevo, Bosnia and Herzegovina; 9 Institute of Cancer and Genomic Sciences, University of Birmingham, Birmingham B15 2TT, UK; 10 National Institute for Health Research (NIHR), Birmingham Biomedical Research Centre, University of Birmingham, Birmingham B15 2TT, UK; 11 Floga Parents Association of Children with Cancer, 11527 Goudi, Greece; 12 Department of Pediatrics, Foundation IRCCS San Gerardo dei Tintori, 20900 Monza, Italy; 13 Department of Medicine and Surgery, University of Milano-Bicocca, 20126 Monza, Italy

**Keywords:** childhood, cancer, malignancy, standards of care, Europe

## Abstract

Cancer in children and adolescents is rare. Therefore, experienced multidisciplinary teams of health care professionals and input from patient advocates are needed. Within the ESCALIER project, we present the current situation of care for children and adolescents with cancer in Europe from the perspective of these stakeholders and highlight the topics relevant for them. A survey developed by representatives from the European Society for Paediatric Oncology (SIOPE) and Childhood Cancer International-Europe (CCI-E) was sent to European paediatric oncologists and patient organizations. We analysed all six questions related to general aspects of care and ten questions especially relevant for SIOPE or CCI-E using descriptive statistics. In total, 159 paediatric oncologists from 35 European countries and 41 CCI-E member organizations from 30 countries replied. Six of the ten questions selected by SIOPE and CCI-E representatives were identical and covered topics from diagnosis to end of treatment and follow-up care. This highlights the alignment of topics relevant for both stakeholders. However, the answers provided by SIOPE and CCI-E respondents to the same questions differed to varying degrees, and answers also differed between respondents from the same country. The differences in the answers provided to our survey highlight the need to raise awareness, improve knowledge, and strengthen communication between different stakeholders, organisations, patients, and families. The stakeholders’ different experiences and knowledge must be considered, and can thus strengthen common goals to provide the best possible care to children and adolescents with cancer in Europe.

## 1. Introduction

Cancer in children and adolescents is rare compared to adults. The incidence rate in Europe is around 177 per million children and 4042 per 100,000 adults [1,2]. As a result, cancer in children and adolescents is less represented among the top priorities of the political agenda of many European countries. In addition, the delivery of care differs between and within countries. In contrast to cancer in adults, survival of children and adolescents with cancer is much higher. The 5-year survival of children and adolescents up to 14 years of age and diagnosed with cancer between 2000 and 2013 in Europe is 81% (95% CI 81–82) [3]. In adults diagnosed with cancer between 2000 and 2007 in Europe, only about one third of all cancers had a 5-year survival rate over 80%, and about one quarter had 5-year survival rates below 30% [4]. Despite the high survival rate, many children and adolescents suffer from late effects of the cancer itself or its treatment [5,6]. The reported proportion of childhood cancer survivors having late effects depends on the treatment era, the treatment received, the time elapsed since treatment, the severity of the late effects, and the method of data collection (self-reported survey versus extraction from medical records), and therefore ranges from around 15% to 99% [5,6,7]. 

Caring for children and adolescents with cancer holds many challenges, partly linked to the unique circumstances related to their young age at diagnosis and the effects on their families. Parents and caregivers become absent from their workplaces, resulting in financial issues, and siblings must be taken care of by alternative caregivers. Furthermore, local conditions in the hospitals need to be adapted to the needs of children and adolescents—clinical examinations and investigations may be prolonged and need more preparation time (e.g., sedation for lumbar puncture or bone marrow biopsy). As life expectancy is high for most children and adolescents after the end of treatment, careful and evidence-based long-term follow-up care and a structured transition into adult care are needed to ensure the highest possible quality of life. All these aspects of care for childhood cancer patients and survivors require many disciplines and stakeholders, including medical disciplines (e.g., paediatric oncology, radiology, surgery), allied health care disciplines (e.g., physiotherapy, nutrition, psychology), and patient advocates and organizations (e.g., Childhood Cancer International–Europe (CCI-E), local and national parent organizations, the European Society for Paediatric Oncology (SIOPE)). The perspective of every single discipline and stakeholder is valuable and needs to be integrated into the care of these children and adolescents. 

SIOPE is the single united European organization for professionals working in the field of childhood cancer (https://siopeurope.eu). SIOPE comprises the Clinical Research Council that brings together the existing European Clinical Trial Groups and the National Paediatric Haematology–Oncology Societies (NaPHOS) from 35 countries across Europe. CCI-E is the largest European childhood cancer patient organisation (https://ccieurope.eu). It comprises 63 childhood cancer parents’ and survivors´ groups and other childhood cancer patient organisations in 34 European countries. Both organizations offer an important platform for exchange and collaboration for their respective members, and foster collaboration across stakeholders. For SIOPE and CCI-E, the vision is to improve survival and quality of care by curing all children and adolescents with cancer through the best possible care and with no or as few late effects as possible.

In 2009, SIOPE initiated a report on the current standards of care within paediatric oncology across Europe. The topics of this report resulted from a survey among European paediatric oncology representatives. The survey results were further discussed during a conference with paediatric oncologists, parent organisations, and policymakers, and resulted in the “European Standards of Care for Children with Cancer” (ESCCC). The aim of this document, consisting of 15 chapters and available in 22 languages, was to act as a tool for professionals and patient representatives to advocate for standards of care across Europe. Given the time elapsed since then, an update was considered necessary due to important advances in diagnostic approaches, treatment options, and additional knowledge on long-term follow-up care in the last 15 years. We therefore adopted a similar approach as for the first document and performed a pan-European survey, sent to paediatric oncologists and patient advocates. Here, we present key aspects from this survey, the ESCALIER (European Standards of CAre for ChiLdren wIth CancER) project. The ESCALIER project aims to map the current situation of care for children and adolescents with cancer in Europe from the paediatric oncologists’ and patient advocates’ perspective. It further aims to highlight differences and areas to be improved in the future, as well as to strengthen collaboration to promote equal access to care. 

## 2. Materials and Methods

In August 2022, the link to an online survey was sent via email to the 32 NaPHOS chairs, representing paediatric oncology professionals, and to all (n = 63) member organisations of CCI-E, representing patient advocates. The patient advocates stand for childhood cancer patients, survivors, and parents. The NaPHOS chairs were asked to forward the survey to one representative of each centre caring for children and adolescents with cancer in their country. It was not feasible to track if and to how many representatives the survey was forwarded by each NaPHOS chair. Therefore, the denominator could not be defined for the analysis. The survey questions were formulated based on the previous ESCCC document, and additional aspects were provided by the board members of SIOPE and CCI-E. The survey consisted of 80 questions, divided into 18 sections: (1) general information, (2) information about the ESCCC document, (3) information about the national structure, (4) information provided to newly diagnosed patients, (5) treatment delivery, (6) clinical trials and innovation, (7) cross-border care, (8) rehabilitation, (9) survivorship, (10) pain management and palliative care, (11) paediatric oncology hospital staff, (12) collaboration between hospitals and local parent/patient organisations, (13) general hospital facilities, (14) hospital facilities for patients, (15) hospital facilities for parents, (16) social and financial burden to families, (17) hospitalised children’s rights, and (18) the rights of parents and family members (Supplemental File S1). The answer options were mainly multiple choice and single answer options; free text fields were optional for some questions to specify options that could not be selected and for additional comments (Supplemental File S1). 

This manuscript reports the analyses of the questions pertaining to the general understanding of national and European childhood cancer care frameworks by including all six general questions about the ESCCC and paediatric oncology on national levels (sections (2) and (3) of the survey). From the remaining 70 questions, SIOPE and CCI-E representatives selected the ten most relevant questions for their community each. Questions with a subjective component (e.g., “in your opinion…”) or specific questions about the ward or hospital (e.g., “What is the age limit for treatment in the paediatric cancer ward you work with”) were excluded because they cannot always be objectively answered by one representative only and most probably not by participants outside the hospital. The difference in respondents from SIOPE (n = 159) and CCI-E (n = 41) allowed descriptive analysis only. We used the software-package “R” (R Core Team, 2022), and maps were created by using mapchart (www.mapchart.net, accessed on 2 July 2023). 

## 3. Results

**Figure 1:**
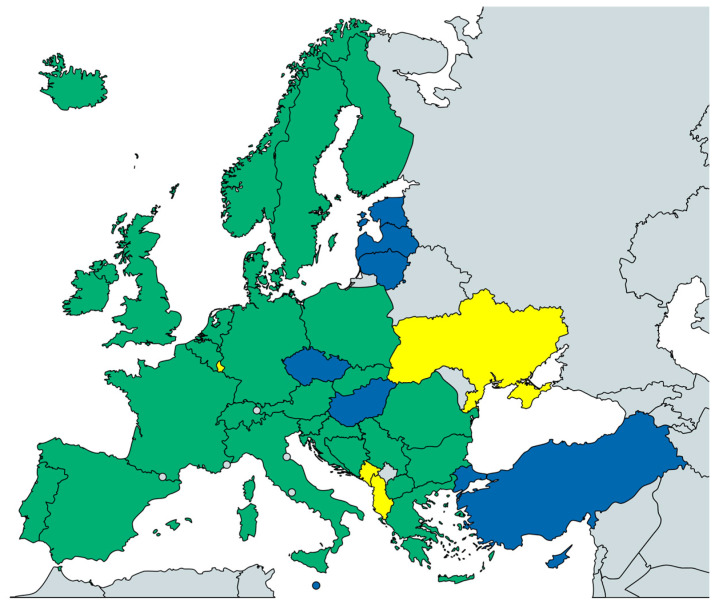
Participating countries in the survey. (green = participants from SIOPE and CCI-Europe; yellow = CCI-E only, blue = SIOPE only).

In total, 159 paediatric oncologists from 35 countries replied (Figure 1, Supplemental File S2). The survey showed that most responding paediatric oncologists (77%) were familiar with the ESCCC, rated the document as helpful (68%), and had used it at least once (61%). The ESCCC was most frequently used for benchmarking standards in the respective centre and to inform other health care professionals (Table 1). Members of 41 CCI-E organizations from 30 countries replied (Figure 1, Supplemental File S2). Most of them (83%) reported being familiar with the ESCCC, rated it helpful (73%), and used it mainly to inform parents, patients, survivors, and health care professionals (Table 1). Paediatric oncologists stated that most children and adolescents with cancer (96%) are included in cancer registries in their countries, with 75% included in childhood cancer registries. In addition, 114 paediatric oncologists (72%) reported that their country has a national cancer plan or equivalent policy document addressing childhood cancer. However, 14% of paediatric oncologists were unsure. The patient advocates’ responses differed for these national topics, with smaller proportions of patient advocates who agreed to the systematic registration of childhood cancer (66%), the registration in childhood cancer registries (44%), and the consideration of childhood cancer in the national cancer plan or equivalent document (59%) (Table 1).

Of the ten questions selected independently by SIOPE and CCI-E representatives, six were identical (Table 1, Supplemental File S3). The questions selected by both stakeholders cover topics through the whole treatment trajectory, from diagnosis to end of treatment and follow-up care, including transition to adult care. Two thirds of paediatric oncologists (66%) agreed on the existence of established procedures to communicate diagnosis and treatment options to patients and their families. Only 41% of patient advocates agreed with this statement and one third (34%) did not know whether established procedures exist. Most paediatric oncologists (98%) reported that clinical trials are accessible in their countries. The questionnaire did not further ask about the type of clinical trial.

Two third (64%) of patient advocates have agreed to the statement about access to clinical trials. Issues in accessing essential medicines were perceived similarly (54% and 58% respectively) by both stakeholders. However, the perception of the frequency of issues in accessing essential medicines differed. Paediatric oncologists reported issues more often than patient advocates. Nearly all paediatric oncologists (99%) and patient advocates (95%) reported that follow-up care is provided in their country, but of varying duration and not in every centre. More paediatric oncologists than patient representatives agreed that a tool for follow-up care (43% vs. 20%) and a transition programme to adult care (46% vs. 37%) exists in their country (Table 1).

**Figure 2:**
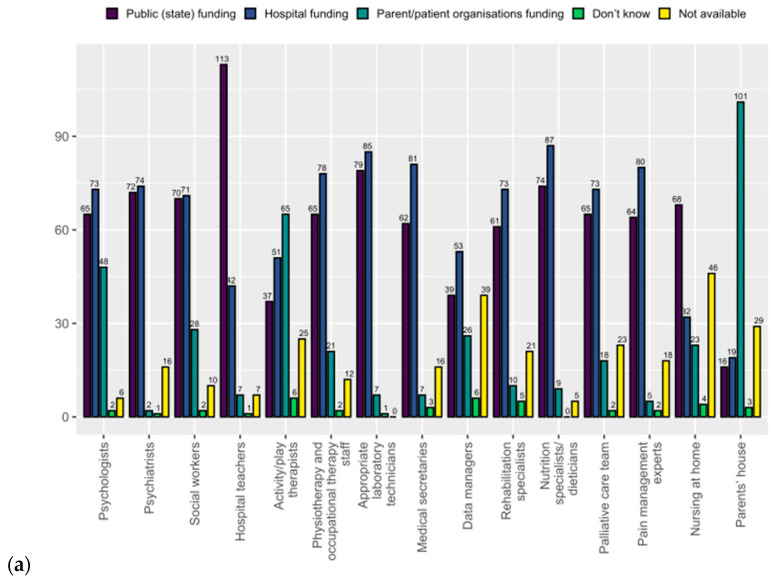

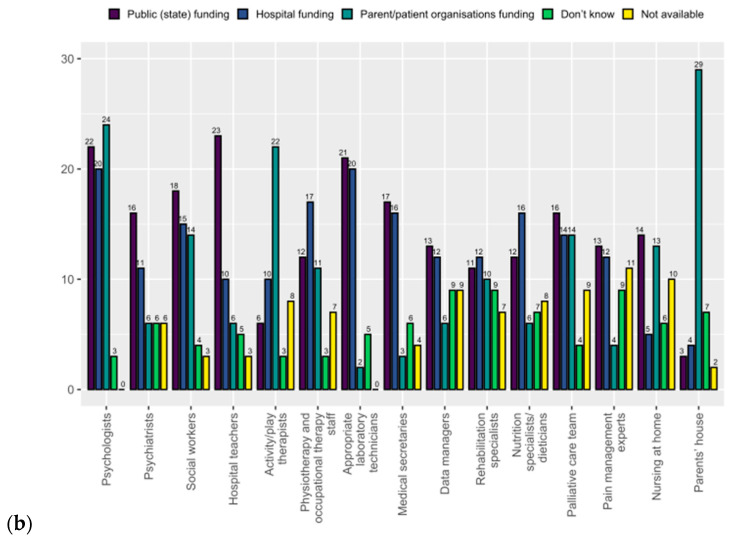
Availability and funding of personnel and related services by SIOPE (**a**) and CCI-Europe (**b**).

The four questions selected by SIOPE representatives only include the interaction between patient advocates and healthcare professionals, availability and funding of specific personnel, supportive systems for patients and families, and coverage of treatment costs. Sixteen paediatric oncologists (10%) reported missing or few interactions with patient advocates, which was not reported by patient advocates themselves (Table 2, Supplemental File S3). Nursing at home (35%), data managers availability (30%), and activity/play therapy (21%) were the top three services reported as unavailable by paediatric oncologists. From patient advocates’ perspective the respective three unavailable services were pain management experts (27%), nursing at home (24%), palliative care team, and data managers (22% each). Except for activity/play therapy (55%) and housing options for parents (82%), services are mainly paid for by public or hospital funds (Figure 2). A support system for patients and families (e.g., help with social, administrative, financial, and legal issues) is reported to be in place by 96% of paediatric oncologists, funded from multiple sources. Paediatric oncologists and patient advocates stated that the treatment costs are mainly covered by public funds (97% vs. 93%).

The four questions selected by CCI-E representatives only include late effects, palliative care, and advocacy. According to the response of 20% of patient advocates, the parents, patients, and survivors are not informed in a timely and age-appropriate manner about possible late effects. This was not stated by paediatric oncologists (Table 2, Supplemental File S3). Although paediatric palliative care service was stated to be available by most patient advocates and paediatric oncologists (80% vs. 84%), the place where palliative care is offered (at home, hospital, or hospice), and the time of the first introduction of the palliative care team differ between the respondents. Paediatric oncologists and patient advocates selected the same top four topics that the European community should advocate for at the European level: establishing legally binding financial compensation for families taking care of an ill child, securing the right to parental leave, requesting flexible working arrangements, and establishing laws on employment protection.

Throughout the survey, the answers differed not only between paediatric oncologists and patient advocates, but also within the same stakeholders from the same country (Supplemental File S4). Even though the differences were more pronounced for some of the questions, we could not identify a pattern (e.g., larger differences in questions about local circumstances than national topics). The proportion of participants answering with “don’t know” was higher for patient advocates than for paediatric oncologists.

## 4. Discussion

This pan-European survey, reflecting the perspectives of paediatric oncologists and patient advocates, provides an overview about the topics perceived most relevant for the current delivery of care for children and adolescents with cancer in Europe. Six out of ten questions were selected by representatives from SIOPE and CCI-E, highlighting that both stakeholders consider the same topics of care important. However, the answers provided differ between the stakeholders and build a basis to work together to educate, raise awareness, and finally contribute to improvement of care and to overcome inequalities.

The answers provided by paediatric oncologists and patient advocates from the same country differed to various degrees. This highlights the urgent need to level up and align the state of knowledge within the different countries. This can only be reached by increasing education, information sharing, awareness, communication, dissemination, and collaboration. Differences between both stakeholder groups from the same country might arise from real differences in daily practice, differences in the perceptions, knowledge, or experiences of the same situation, or how the questions were formulated. Questions about local practices (e.g., “When is palliative care integrated into the care pathway in your centre?”) might be difficult to answer for patients advocates, especially for countries with more than one centre. In addition, due to the smaller sample size of patient advocates, their individual answers carry more weight than the answers from individual paediatric oncologists. Having different priorities might be another reason for discrepancies between both stakeholders. Paediatric oncologists might be more focused on the treatment, the management of acute toxicities, or other somatic or psychological problems, and might underestimate worries and fears crucial to patient advocates. Regardless of the reasons for the differences, the identified topics can be taken as starting points for the education of paediatric oncologists and patient advocates on topics that are important either to both or one of the stakeholder groups. In this context, communication between paediatric oncologists and patient advocates is crucial and should be promoted at all levels (e.g., through joint exchange at the local/hospital level, regional/national meetings, or workshops). 

Differences in answers provided by paediatric oncologists from the same country could be due to insufficient awareness and training (e.g., if childhood cancer is addressed in national cancer plans) or to different daily practices between hospitals (e.g., follow-up care implementation). For both types of differences, harmonisation would be desirable. Different approaches in everyday clinical practice to some extent cannot be completely avoided, nor would it be expected. However, certain jointly agreed regional and national standards (e.g., follow-up care must be available, but exact place of care or duration until transition can differ) must be in place by an implementation process based on local common practices.

Different responses between countries might result from differences in health care systems, for example, reflected by various funding sources to cover treatment costs or the inclusion of childhood cancer in national cancer strategies. The important topic of national cancer strategies and the inclusion of paediatric cancer is currently elaborated on and mapped within the policy pillar of SIOPE—in the “Paediatric cancer in National Cancer Control Plans” project [8]. The results from this project have recently been published [9]. The issue of access to anticancer medicines for children and adolescents with cancer in Europe was elaborated on in 2021 for 68 selected medicines [10]. However, the question about access to anticancer medicines in this publication was part of a larger survey and was asked to clinicians and pharmacists only. Our results add to this publication the overall perception of issues in accessing essential medicines in general. Our results further took the patient advocates’ perspective into account.

In July 2019, the directive on better work-life balance for parents and carers was published in the official journal of the EU [11]. This directive includes a section on carers’ leave, indicating that each person has the right to carers’ leave of five working days per year. However, five days is clearly not enough for parents when their child is diagnosed with cancer, as the treatment can last from 3 months to multiple years. The political topics highlighted in the survey (e.g., legally binding financial compensation for families, right to parental leave, and flexible working arrangements) can only be reached through a joint European effort and one joint voice.

The strength of this study is the pan-European approach with participants from 38 countries and the joint effort between paediatric oncologists and patient advocates in the setup and analysis of the data. Asking the same questions to paediatric oncologists and patient advocates is a strength in some aspects and a limitation in others. Some questions were not formulated to be answered by respondents not working in paediatric oncology service. Here, discrepancies between paediatric oncologists and patient advocates must be interpreted cautiously. In addition, as in every questionnaire-based study, the results from the ESCALIER project harbour the inevitable risk of bias; reporting bias, recall bias, sampling bias, or social desirability bias. For some questions, a subjective influence on the answers cannot be excluded (e.g., difficult for one or few representatives per country to judge when palliative care is integrated into care), and in some cases, we did not verify the accuracy of the views in the answers (e.g., whether cancer in children is included in the national cancer plans). The ESCALIER project aimed to describe the current situation of care for children and adolescents with cancer in Europe from a broader perspective. Therefore, we asked for examples of whether support systems are in place, but did not ask about the exact support system or its success. Even though 38 European countries are covered by this survey, only 159 paediatric oncologists responded. As we did not ask the NaPHOS chairs to report the number of paediatric oncologists they sent the survey to, we have no information on the denominator. Some countries are relatively overrepresented (e.g., Turkey), whilst others were underrepresented (e.g., Germany) based on the size of the country and the number of hospitals treating children with cancer. For CCI-E, only 41 organisations responded, and this figure might not represent the needs and views of all European childhood cancer patient organizations. For future questionnaires, it could also be helpful to translate them into their respective national language.

## 5. Conclusions

Our results highlight important areas where SIOPE and CCI-E need to take action in the future. It is crucial to raise awareness and improve knowledge and communication between different stakeholders, including paediatric oncologists, other disciplines involved in the care of children and adolescents with cancer, patient advocates, organisations, and families. The different experiences and knowledge from various stakeholders unite and strengthen joint efforts to overcome inequalities in the treatment of children and adolescents with cancer in Europe. Results from this survey will be used for the updated ESCCC and could stimulate education, communication, and collaboration on national and European levels.

## Figures and Tables

**Table 1:** Answers provided by SIOPE and CCI-E to the selected general questions and questions selected by SIOPE and CCI-Europe.

	SIOPE (n = 159) n (%)	CCI-E (n = 41) n (%)
**General questions**		
Are you familiar with the Standards of Care?	Yes: 122 (77) No: 37 (23)	Yes: 34 (83) No: 7 (17)
Have you ever used the European Standards of Care for Children with Cancer? (multiple answer options)	Yes, for political leverage: 27 (17) Yes, for benchmarking standards in the centre: 65 (41) Yes, to inform parents/patients/survivors: 39 (25) Yes, to inform healthcare professionals: 49 (31) No: 58 (36) Yes, for other reasons (please explain): 6 (4)	Yes, for political leverage: 11 (27) Yes, for benchmarking standards in the centre: 6 (15) Yes, to inform parents/patients/survivors: 15 (37) Yes, to inform healthcare professionals: 14 (34) No: 15 (37) Yes, for other reasons (please explain): 3 (7)
	At least one yes-answer: 97 (61)	At least one yes-answer: 62 (63)
Do you find the European Standards of Care for Children with Cancer helpful?	Not at all: 1 (1) Rather not: 4 (3) Don’t know: 44 (28) Quite helpful: 66 (42) Very helpful: 41 (26)	Not at all: 0 Rather not: 1 (2) Don’t know: 10 (24) Quite helpful: 13 (32) Very helpful: 17 (41)
Is there a national society of healthcare professionals in paediatric oncology in your country?	Yes: 152 (96) No: 6 (4) Don’t know: 1 (1)	Yes: 28 (68) No: 8 (20) Don’t know: 5 (12)
Are childhood cancer cases systematically registered at the national level in your country?	Yes, in the national childhood cancer registry: 116 (73) Yes, in the overall national cancer registry: 37 (23) No: 5 (3) Don’t know: 1 (1)	Yes, in the national childhood cancer registry: 18 (44) Yes, in the overall national cancer registry: 9 (22) No: 10 (24) Don’t know: 4 (10)
Is childhood cancer addressed in your country’s national cancer plan or equivalent policy document?	Yes: 114 (72) No: 23 (14) Don’t know: 22 (14)	Yes: 24 (59) No: 13 (32) Don’t know: 4 (10)
**Questions selected by SIOPE and CCI-Europe**		
Is there a procedure (e.g., guidelines, best practices) established by healthcare professionals to communicate diagnosis and treatment options to the patient and their family?	Yes: 105 (66) No: 51 (32) Don’t know: 3 (2)	Yes: 17 (41) No: 10 (24) Don’t know: 14 (34)
Are clinical trials accessible in your hospital/country?	Yes, it is a standard: 99 (62) Yes, but limited: 57 (36) No: 3 (2) Don’t know: 0	Yes, it is a standard: 15 (37) Yes, but limited: 11 (27) No: 11 (27) Don’t know: 4 (10)
Do you face issues in accessing essential medicines for childhood cancer in your country?	Yes, frequently: 34 (21) Yes, sometimes: 52 (33) No: 72 (45) Don’t know: 1 (1)	Yes, frequently: 3 (7) Yes, sometimes: 21 (51) No: 16 (39) Don’t know: 1 (2)
Is medical follow-up provided to childhood cancer survivors in your country?	Yes, for a lifetime: 63 (40) Yes, for up to 5 years: 14 (9) Yes, for up to 10 years: 47 (30) No: 1 (1) Other: 34 (20) *	Yes, for a lifetime: 12 (29) Yes, for up to 5 years: 13 (32) Yes, for up to 10 years: 6 (15) No: 2 (5) Other: 8 (20) °
Does a tool for follow-up care exist in your country (e.g., Survivorship Passport)?	Yes: 69 (43) No: 85 (53) Don’t know: 5 (3)	Yes: 8 (20) No: 29 (71) Don’t know: 4 (10)
Is there a programme for transitioning from paediatric to adult healthcare in your country?	Yes: 73 (46) No: 75 (47) Don’t know: 11 (7)	Yes: 15 (37) No: 22 (54) Don’t know: 4 (10)

* “Other”: all 34 answers included some form of follow-up care, including the following answers: for up to 18 years of age; for up to 20–24 years of age; time of follow-up in paediatric oncology depends on the risk group/risk-stratified; depending on age at diagnosis for more than 10 years bit not a lifetime; ° “Other”: all eight answers included some form of follow-up care.

**Table 2:** Answers provided by SIOPE and CCI-E to the questions selected by SIOPE or CCI-Europe only.

**Questions Selected by SIOPE Only**		
How frequent is the interaction between parent/patient organisation(s) and healthcare professionals in your paediatric oncology centre?	Never: 6 (4) Very rare: 10 (6) Occasional: 36 (23) Very frequent: 81 (51) Daily: 26 (16)	Never: 0 Very rare: 0 Occasional: 14 (34) Very frequent: 17 (41) Daily: 10 (24)
Which of the following personnel and related services are available and who funds them?	See Figure 2a	See Figure 2b
Is there a support system in place for patients and their families, e.g., to help them with administrative, financial, and legal issues, advise them when the first diagnosis is made?	Yes, provided by the hospital: 77 (48) Yes, provided by the parent/patient organisation(s): 42 (26) Yes, as joint initiative: 57 (36) No: 7 (4) Don’t know: 1 (1) Other: 13 (8)	Yes, provided by the hospital: 15 (37) Yes, provided by the parent/patient organisation(s): 20 (49) Yes, as joint initiative: 15 (37) No: 5 (12) Don’t know: 0 Other: 5 (12)
	At least one yes-answer: 152 (96)	At least one yes-answer: 36 (88)
How are the treatment costs covered in your country?	From the state: 154 (97) From private insurance: 16 (10) From parent/patient organisation(s): 10 (6) Other: 8 (5)	From the state: 38 (93) From private insurance: 8 (20) From parent/patient organisation(s): 9 (22) Other: 5 (12)
	Combination of state and others: 154 (97) Private and others: 5 (3)	Combination of state and others: 38 (93) Other options than state: 3 (7)
**Questions Selected by CCI-Europe Only**		
Are parents/patients/survivors informed about possible late effects in a timely and age-appropriate manner?	Yes, always: 113 (71) Yes, in most cases: 44 (28) No: 0 Don’t know: 2 (1)	Yes, always: 4 (10) Yes, in most cases: 23 (56) No: 8 (20) Don’t know: 6 (15)
Are there any paediatric palliative care services available in your centre?	Yes, at home: 103 (65) Yes, in the hospital: 115 (72) Yes, in the hospice: 38 (24) No: 25 (16) Don’t know: 0	Yes, at home: 24 (59) Yes, in the hospital: 24 (59) Yes, in the hospice: 13 (32) No: 9 (22) Don’t know: 1 (2)
	Any yes: 134 (84)	Any yes: 33 (80)
When is palliative care integrated into the care pathway in your centre?	At diagnosis: 19 (12) During treatment: 35 (22) In relapse: 33 (21) In the presence of terminal illness: 66 (42) Don’t know: 1 (1) Not applicable: 5 (3)	At diagnosis: 2 (5) During treatment: 4 (10) In relapse: 3 (7) In the presence of terminal illness: 23 (56) Don’t know: 4 (10) Not applicable: 5 (12)
In your opinion, for which aspects should we advocate in the framework of carers’ rights at the European level? (additional point for discussion put forward by CCI-E)	Secure right to parental leave: 113 (71) Secure right to request flexible working arrangements: 113 (71) Establish law on employment protection: 107 (67) Establish legally binding financial compensation for families taking care of an ill child: 106 (67) Ensure formal service provision to support families during their carers journey: 87 (55) Raise awareness on the needs of carers rights: 75 (47) Educating carers on their rights: 70 (44) Formalise informal care at national levels: 65 (41) Other (please specify): 7 (4)	Secure right to parental leave: 31 (76) Secure right to request flexible working arrangements: 27 (66) Establish law on employment protection: 30 (73) Establish legally binding financial compensation for families taking care of an ill child: 34 (83) Ensure formal service provision to support families during their carers journey: 22 (54) Raise awareness on the needs of carers rights: 26 (63) Educating carers on their rights: 21 (51) Formalise informal care at national levels: 21 (51) Other (please specify): 2 (5)

## Data Availability

Data are available upon request to the authors.

## Supplementary Materials

The following supporting information can be downloaded at: https://www.mdpi.com/article/10.3390/curroncol32020084/s1, Supplemental File S1: Questionnaire sent to the participants; Supplemental File S2: Countries represented in the questionnaire; Supplemental File S3: Detailed answers to the selected questions by SIOPE and CCI-E representatives; Supplemental File S4: Two example answers stratified by country.

## Abbreviations

The following abbreviations are used in this manuscript:

CCI-E	Childhood Cancer International—Europe
CCS	Childhood Cancer Survivors
ESCALIER	European Standards of Care for Children with Cancer
ESCCC	European Standards of Care for Children with Cancer
SIOPE	European Society for Paediatric Oncology
