# Supplementary material for: The Healthcare Professionals’ and Patient Advocates’ Perspectives on the Care for Children with Cancer in Europe—A Report from the ESCALIER Project
Source: Curr Oncol. 2025 Jan 31;32(2):84. doi: 10.3390/curroncol32020084 (PMC11854544; doi:10.3390/curroncol32020084)

The Healthcare Professionals' and Patient Advocates' Perspectives on the Care for Children with Cancer in Europe—A Report from the ESCALIER Project

Maria Otth, Marko Ocokoljic, Theodora Armenkova, Irina Ban, Samira Essiaf, Maximilian Hopfgartner, Lejla Kameric, Pamela R. Kearns, Georgia Kokkinou, Carmelo Rizzari, Carina Schneider and Katrin Scheinemann

Supplemental material

Supplemental S1: Questionnaire sent to the participants

Who do you represent?
- Healthcare/research professionals Parent/patient organization
- Please state the name of the hospital/institution you represent:

How would you define your hospital/institution:
- Public paediatric hospital
- Public comprehensive cancer centre (treating both children and adults) Other (please specify)

Benefits of the Document
Are you familiar with the European Standards of Care for Children with Cancer?
- Yes
- No

Have you ever used the European Standards of Care for Children with Cancer?
- Yes, for political leverage
- Yes, for benchmarking standards in the centre Yes, to inform parents/patients/survivors
- Yes, to inform healthcare professionals No
- Yes, for other reasons (please explain)

Do you find the European Standards of Care for Children with Cancer helpful?
- Very helpful
- Quite helpful
- Don’t know
- Rather not
- Not at all

Paediatric Oncology at the National Level
Is there a national society of healthcare professionals in paediatric oncology in your country?
- Yes
- No
- Don't know
- Any comments (optional)

Are childhood cancer cases systematically registered at the national level in your country?
- Yes, in the national childhood cancer registry
- Yes, in the overall national cancer registry
- No
- Don't know
- Any comments (optional)

Are childhood cancer registered using the International Classification of Childhood Cancer ver.3 – ICCC-3?
- Yes
- No
- Don't know
- Not applicable
- Any comments (optional)

Is childhood cancer addressed in your country's national cancer plan or equivalent policy document?
- Yes
- No
- Don't know

Collaboration between Hospitals and Local Parent/Patient Organisations
Are patient/parent organisations in your hospital provided with directions through signage/labelling (e.g. banner, roll-up, poster)?
- Yes
- No
- Don't know

Is there a dedicated room for the parent/patient organisation inside your hospital?
- Yes, a dedicated room for the parent/patient organisation
- Yes, shared room with other entities (e.g. multi-purpose)
- No
- Other (please specify)

How frequent is the interaction between parent/patient organisation(s) and healthcare professionals in your paediatric oncology centre?
- Daily
- Very frequent
- Occasionally
- Very rare
- Never

Do professionals in the hospital receive professional training/education abroad?
- Yes, at regular time intervals (e.g. once every year)
- Yes, on an ad hoc basis
- No
- Don't know
- Any comments (optional)

Do professionals receive continuous professional training/education in your country?
- Yes, at regular time intervals (e.g. once every year)
- Yes, but not systematically
- No
- Don't know
- Any comments (optional)

Who covers the educational cost for professionals?
- Public authorities Hospital/institution
- Medical professionals themselves Parent/patient organisations
- Don't know

General Hospital Facilities
Which of the following personnel and related services are available and who is funding them?
(answer options: public state, hospital, parent/patient organisations, don't know, not available)
- Psychologists
- Psychiatrists
- Social workers

- Hospital teachers
- Activity/play therapists
- Physiotherapy and occupational therapy staff
- Appropriate laboratory technicians
- Medical secretaries
- Data managers
- Rehabilitation specialists
- Nutrition specialists/ dieticians
- Palliative care team
- Pain management experts
- Nursing at home
- Parents' house
- Other (please specify)

Hospital Facilities for Patients

Are there single or at most two-bedded rooms in your hospital?
- Yes, it is a standard
- Yes, in most cases
- Yes, in some cases
- No

Are those hospital rooms equipped with their own bathroom and toilet?
- Yes, it is a standard
- Yes, in most cases
- Yes, in some cases
- No
- Any other comments (optional)

Are there separate facilities for different age groups (e.g. pre- and post-puberty)?
- Yes, it is a standard
- Yes, in most cases
- Yes, in some cases
- No
- Any other comments (optional)

Are there living spaces for patients (e.g. common room, play area)?
(answer options: yes, no, don't know)
- Only haemato-oncology
- Shared with other paediatric patients
- Open to siblings
- Open to parents
- Open to animators/ volunteers

Are there education provisions and arrangements for children while in treatment?
(answer options: yes, no, don't know)
- Pre-school
- Primary school
- Secondary school
- Provided in patient's room
- Provided in a dedicated space/classroom
- Organised by hospital
- Organised by parents/patient
- Organised by parent/patient organisations

- Any other comments (optional)

Do healthcare professionals support parents in making a joint decision to reintegrate their child to school and social life post-treatment?
- Yes, always
- Yes, mostly
- Yes, rarely
- No

Hospital Facilities for Parents
Is there a bed available for a parent in the patient's room in your hospital?
- Yes, it is a standard
- Yes, in most cases
- Yes, in some cases
- No
- Any other comments (optional)

Is the bed for the parent restricted to only mothers/female caregivers?
- Yes
- No
- Don't know
- Not applicable

Information Provision to Newly Diagnosed Patients
Is there a dedicated space to inform and hold a medical discussion with newly diagnosed patients and parents?
- Yes
- No
- Don't know

Are the hospitalised child's rights displayed in the ward?
- Yes
- No
- Don't know

Do patients and parents receive information about their rights?
- Yes
- No
- Don't know

Is there a procedure (e.g. guidelines, best practices) established by healthcare professionals to communicate diagnosis and treatment options to the patient and their family?
- Yes
- No
- Don't know

In your opinion, are patients/parents adequately informed of cancer diagnosis and treatment procedures in your hospital (e.g. late-effects, treatment modalities, fertility preservation)?

- Yes, always
- Yes, in most cases
- Yes, in some cases
- No
- Don't know
- Any comments (optional)

Is there a dedicated healthcare professional for the patient's and parent's further questions on the treatment journey?

- Yes
- No
- Don't know

Are parent/patient organisations informed by the hospital of newly diagnosed patients to approach and help families?

- Yes, always
- Yes, in most cases
- Yes, in some cases
- Don't know
- No

Is there a support system in place for patients and their families, e.g. to help them with social, administrative, financial, and legal issues, advise them when the first diagnosis is made?

- Yes, provided by the hospital
- Yes, provided by the parent/patient organisation(s)
- Yes, as a joint initiative by both, parent/patient organisation and hospital No
- Don't know
- Other (please specify)

Hospitalised Child's Rights

What is the age limit for treatment in the paediatric cancer ward you work with?

- Up to 15 y.o.
- Up to 16 y.o.
- Up to 17 y.o.
- Up to 18 y.o.
- 18+ y.o.
- Other (please specify)

Do you consider that the following rights of hospitalised children, adolescents and young adults are respected in the hospital you work in/with?
(answer options: Yes, always, Yes, mostly, Yes, but only rarely, No)

- Constant and continuous parental involvement
- Adequate accommodation for parents in the hospital
- Play and education facilities
- ge-appropriate environment
- A multidisciplinary treatment team
- The right to adequate information
- The right to continuity of care
- Appropriate pain management
- The right to privacy

Social and Financial Burden in Families
How are the treatment costs covered in your country?
- From the state
- From the private insurance
- From parent/patient organisation(s)
- Other (please specify)

Is there financial support for parents' loss of income/absence from work?
- Yes, from the state
- Yes, from parent/patient organisation(s)
- Yes, from other NGOs
- No
- Other (please specify)

How are the parents informed about available funding streams?
- Public authorities
- Healthcare professionals
- Parent/patient organisations
- Social workers at the hospital Brochures
- Other (please specify)

Treatment Delivery
Are you aware of the list of essential medicines for childhood cancer (soon) to be published by SIOP Europe in partnership with CCI-E?
- Yes
- No

Do you face issues in accessing essential medicines for childhood cancer in your country?
- Yes, frequently
- Yes, sometimes
- No
- Don't know

Are you aware of the patients who have been using complementary therapies (e.g. yoga, acupuncture, meditation, music, massage, sports)?
- Yes
- No
- Don't know

Do you find the complementary therapies beneficial to recommend to patients on a regular basis?
- Yes
- No
- Depends on the circumstance
- Don't know

Clinical Trials and Innovation
Are clinical trials accessible in your hospital/country?
- Yes, it is a standard
- Yes, but limited
- No
- Don't know

Is it common practice in your hospital to discuss with parents/patients about taking part in clinical trials?
- Yes
- No
- Don't know

Are patients and/or parents satisfied with the explanations given before signing the assent or consent form?
- Yes
- No
- Don't know
- Not applicable

Are parents and patients informed about the clinical trial progress on a regular basis?
- Yes
- Yes, but only upon request
- No
- Don't know
- Not applicable
- Other (please specify)

Cross-Border Care

Are you familiar with the European Reference Network on Paediatric Cancer (ERN PaedCan)?
- Yes
- No

Does your hospital participate in the ERN PaedCan?
- Yes
- No
- Don't know
- Not applicable (country is not member of EU)

Are patients in your country referred to hospitals abroad?
- Yes, when certain treatments are not available (but not for clinical trials)
- Yes, when certain treatments are not available (including clinical trials)
- No
- Don't know

Who covers the additional costs for treatment abroad (e.g. accommodation, food, documentation, translation)?
- State
- Private health insurance
- Parent/patient organisations
- Families themselves
- Other (please specify)

Rehabilitation
According to the World Health Organization: “rehabilitation is a process aimed at enabling patients to reach and maintain their optimal physical, sensory, intellectual, psychological and social functional level”.

Are there any physiotherapy units in your centre?
- Yes, in the same building
- Yes, near the hospital
- Yes, near the patient's home
- Yes, in a remote location
- Only abroad
- No
- Don't know

Are there psycho-social services to facilitate patient's return to everyday life?
- Yes, provided by the state
- Yes, provided by NGOs No
- Don't know
- Other (please specify)

Are rehabilitation services also available after the patient's treatment?
- Yes, provided by the state
- Yes, provided by NGOs
- No
- Don't know
- Other (please specify)

To whom are rehabilitation services provided to?
- To children and adolescents with cancer only
- To survivors
- To siblings
- To the entire family To other relatives Not provided

Survivorship, Follow up Care, Late Outcomes
Is medical follow-up provided to childhood cancer survivors in your country?
- Yes, for a lifetime
- Yes, for up to 5 years
- Yes, for up to 10 years
- No
- Other (please specify)

Are parents/patients/survivors informed about possible late effects in a timely and age-appropriate manner?
- Yes, always
- Yes, in most cases
- No
- Don't know
- Any comments (optional)

Does a tool for follow-up care exist in your country (e.g. Survivorship Passport)?
- Yes
- No
- Don't know

Who provides follow-up care services in your country?
- Paediatric cancer ward
- Paediatric hospital
- Dedicated follow-up care centre
- General hospital
- Other (please specify)

Does the provided follow-up care adhere to the international follow-up care guidelines (https://www.ighg.org/guidelines/)?
- Yes
- No
- Don't know
- Not applicable

Is there a programme for transitioning from paediatric to adult healthcare in your country?
- Yes
- No
- Don't know
- Any comments (optional)

Pain Management and Palliative Care

Do children and adolescents receive sedation for painful procedures in your centre (e.g. bone marrow aspiration)?
- Yes, always
- Yes, sometimes
- No
- Don't know

Is medical staff attentive to cancer pain in your centre?
- Yes, always
- Yes, sometimes
- No
- Don't know

Are there any paediatric palliative care services available in your centre?
- Yes, at home
- Yes, in the hospital
- Yes, in the hospice
- No
- Don't know

When is palliative care integrated into the care pathway in your centre?
- At diagnosis
- During treatment
- In relapse
- In the presence of terminal illness
- Don't know
- Not applicable

Who runs the palliative care services in your centre?
- State
- Parent/patient organisations
- Other NGOs
- There is no palliative care service available
- Don't know

Rights of Informal Carers (Parents and Family Members)
Which family members mostly take care of the child during their treatment journey?
- Father
- Mother
- Siblings
- Grandparents
- Close relatives (e.g. aunts, uncles)
- Other (please specify)

Are parents entitled to take sick leave days when their child is undergoing treatment?
- Yes
- No
- Don't know

If yes, how many days are they allowed to take?

Is the law protecting parents' jobs when they are absent due to their child's serious illness?
- Yes
- No
- Don't know
- Any comments (optional)

Is there social and/or health insurance in place for informal caregivers (e.g. parents) who need to leave their jobs to provide care for their severely ill child?
- Yes
- No
- Don't know

Which financial compensation streams are provided to informal carers (e.g. parents) who need to leave their jobs to provide care for their severely ill child?
- Dedicated government funding
- National insurance agency funding
- Parent/patient organisations funding
- Charitable grants
- None is being provided
- Other

Do you think that the EU Work-Life Balance Directive can help improve standards of care for childhood cancer patients and their families?
- Yes, definitely
- Yes, probably
- No
- Don't know
- Never heard of the Directive
- Not applicable (country is not an EU Member State)

Do you foresee a need for having specifically tailored education/training for informal carers (e.g. parents) who take care of their ill child?
- Yes
- No
- Don't know

Do you foresee benefits in officially defining informal care through formalisation and recognition at the national level?

- Yes
- No
- Don't know

In your opinion, for which aspects should we advocate in the framework of carers' rights at the European level?

- Secure right to parental leave
- Secure right to request flexible working arrangements
- Establish law on employment protection
- Establish legally binding financial compensation for families taking care of an ill child
- Ensure formal service provision to support families during their carers journey
- Raise awareness on the needs of carers rights
- Educating carers on their rights
- Formalise informal care at national levels
- Other (please specify)

Supplemental S2: countries represented in the questionnaire

SIOPE / Health care professionals	CCI-E / Patient advocates
Austria	Albania
Belgium	Austria
Bosnia and Herzegovina	Belgium
Bulgaria	Bosnia and Herzegovina
Croatia	Bulgaria
Cyprus	Croatia
Czech republic	Denmark
Denmark	France
Estonia	Finland
France	Germany
Finland	Greece
Germany	Ireland
Greece	Italy
Hungary	Iceland
Ireland	Luxembourg
Italy	Macedonia
Iceland	Montenegro
Latvia	Netherlands
Lithuana	Norway
Macedonia	Poland
Malta	Portugal
Netherlands	Romania
Norway	Sweden
Poland	Switzerland
Portugal	Slovenia
Romania	Slovakia
Sweden	Spain
Switzerland	Serbia
Slovenia	United Kingdom
Slovakia	Ukraine
Spain	
Serbia	
Turkey	
United Kingdom	

Supplemental S3: Detailed answers to the selected questions by SIOPE and CCI-E representatives

Is there a procedure (e.g. guidelines, best practices) stablished by healthcare professionals to communicate diagnosis and treatment options to the patient and their family?

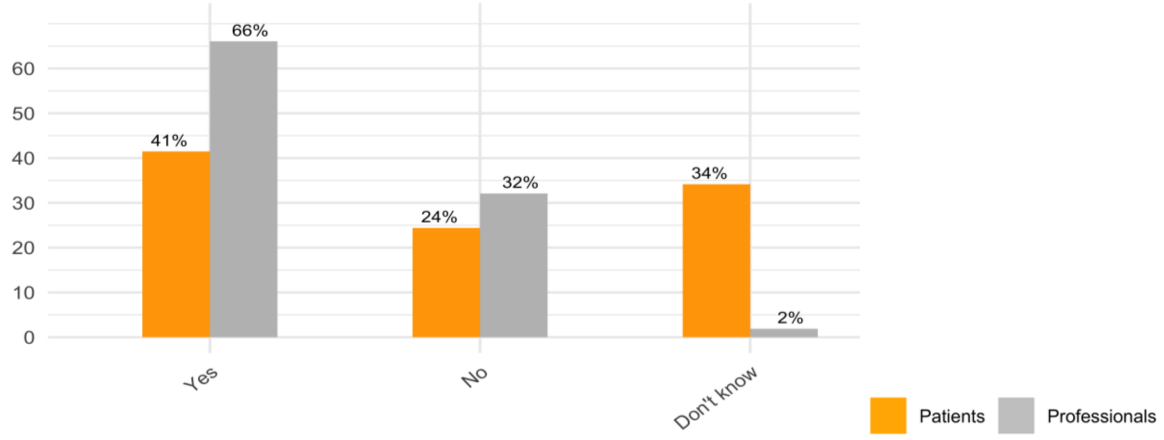

Are clinical trials accessible in your hospital/country?

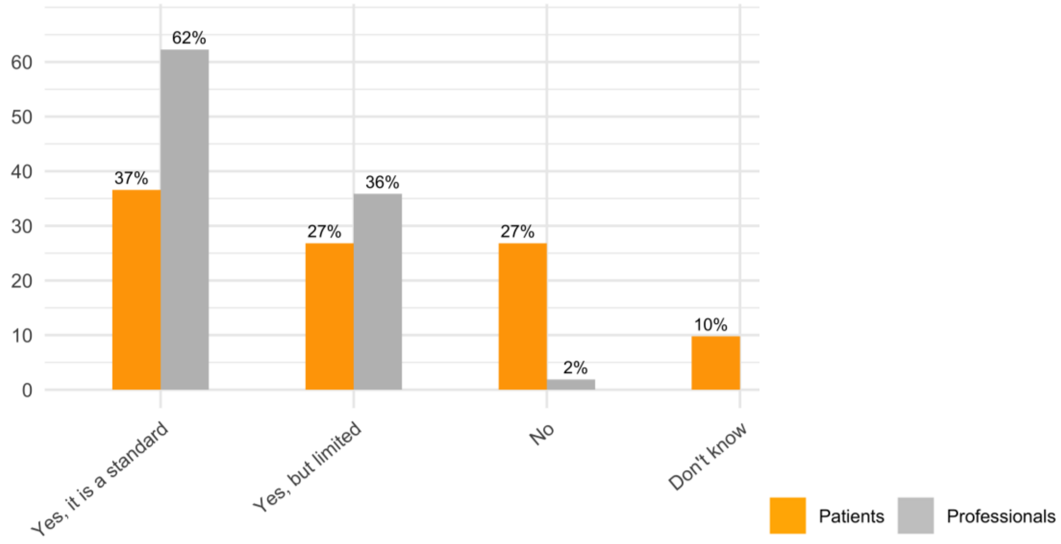

Do you face issues in accessing essential medicines for childhood cancer in your country?

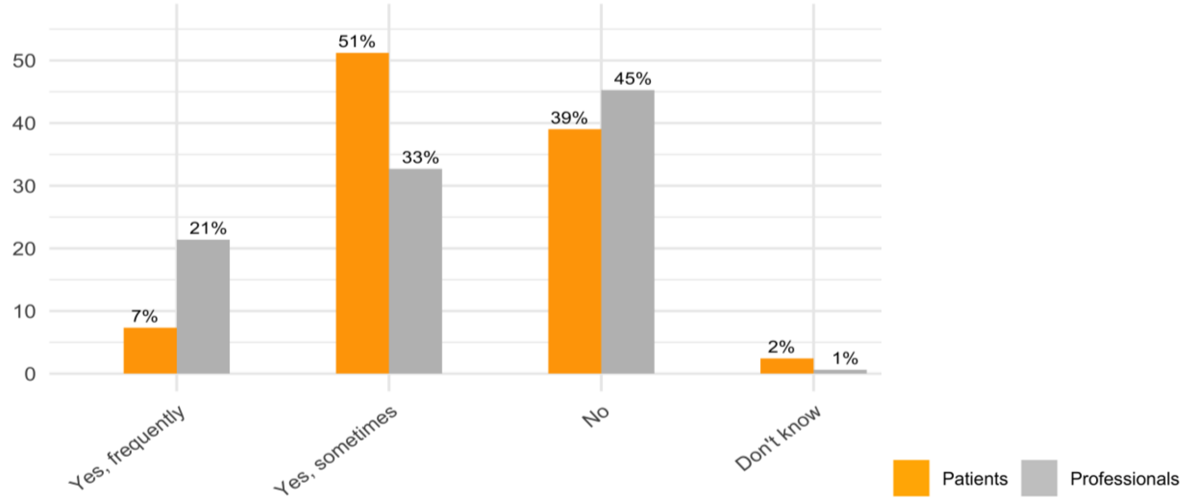

Is medical follow-up provided to childhood cancer survivors in your country?

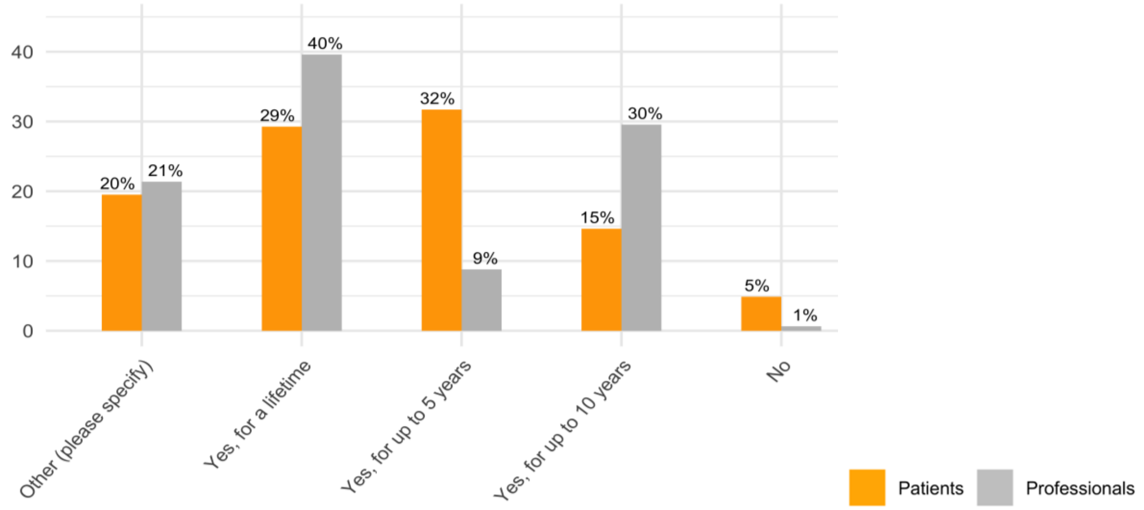

Does a tool for follow-up care exist in your country (e.g. Survivorship Passport)?

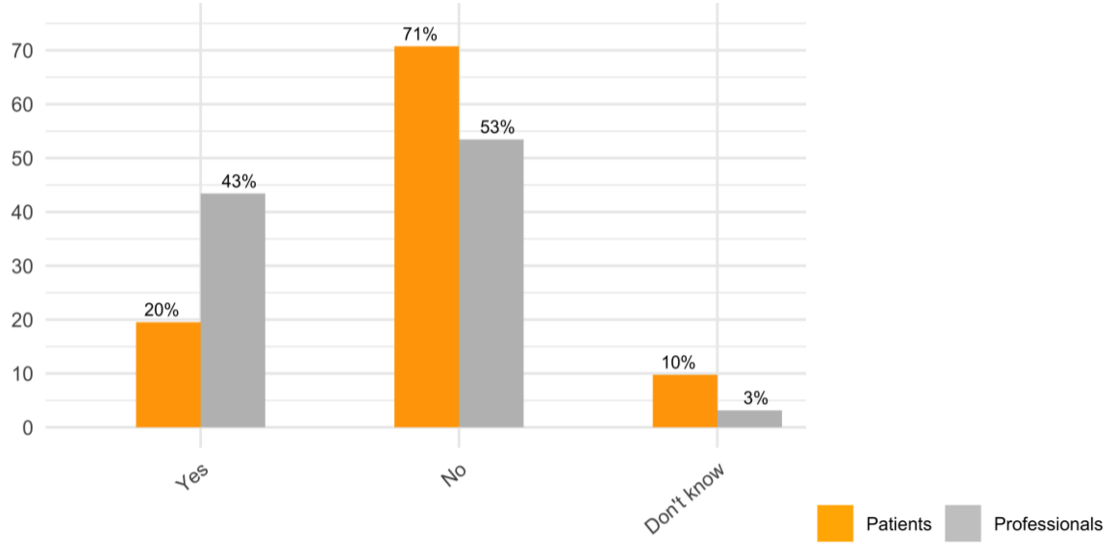

Is there a programme for transitioning from paediatric to adult healthcare in your country?

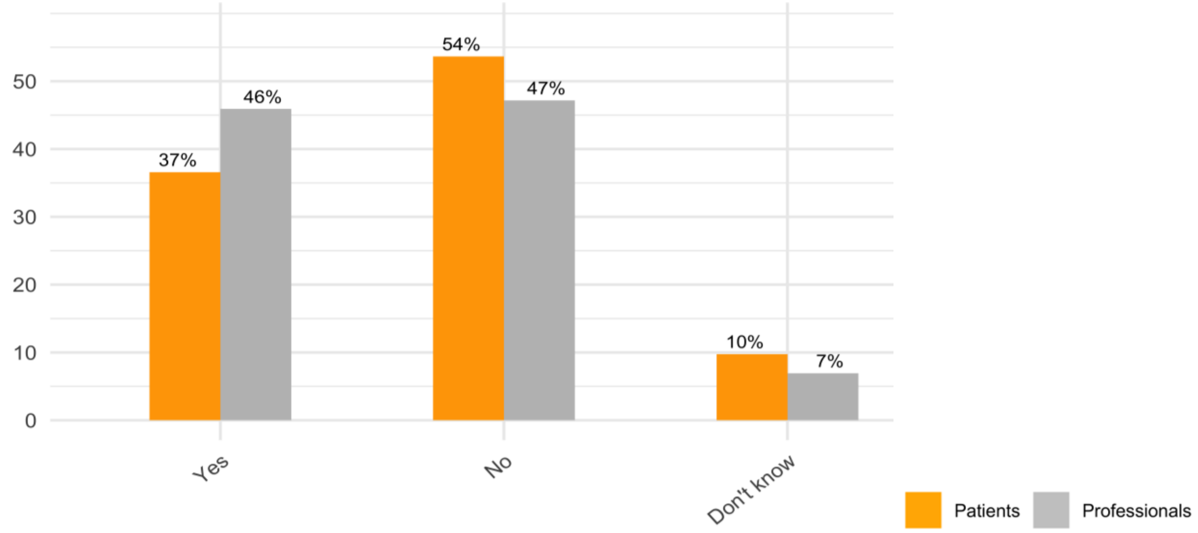

How frequent is the interaction between parent/patient organisation(s) and healthcare professionals in your paediatric oncology centre?

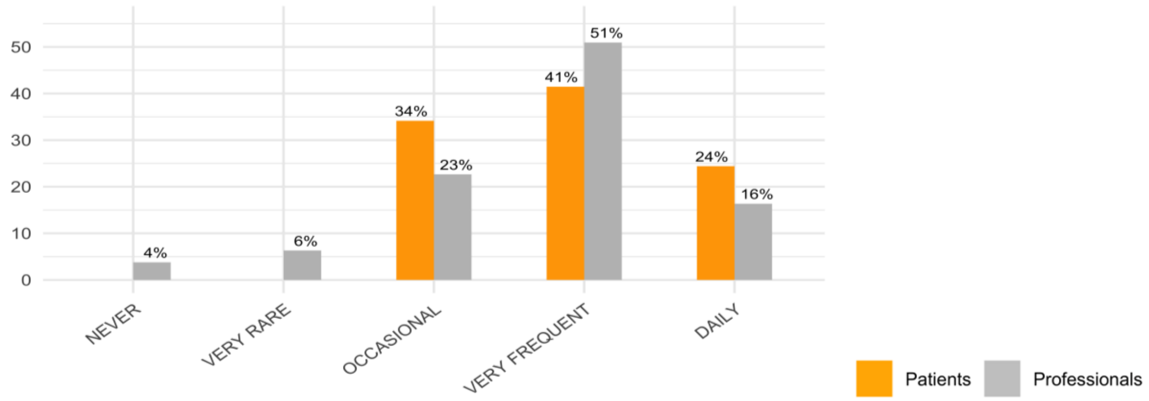

Is there a support system in place for patients and their families, e.g. to help them with ESCCCial, administrative, financial, and legal issues, advise them when the first diagnosis is made?

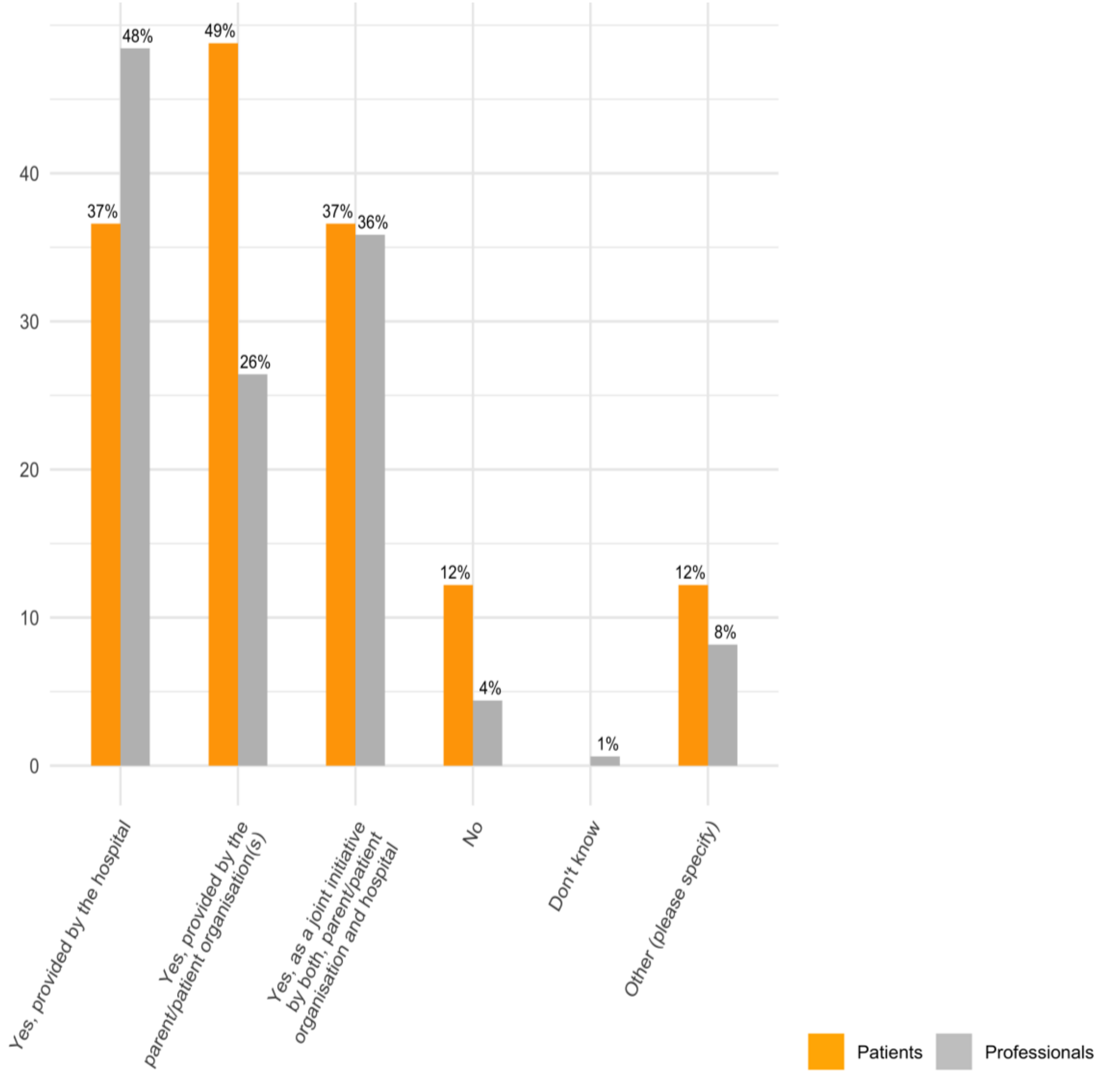

How are the treatment costs covered in your country?

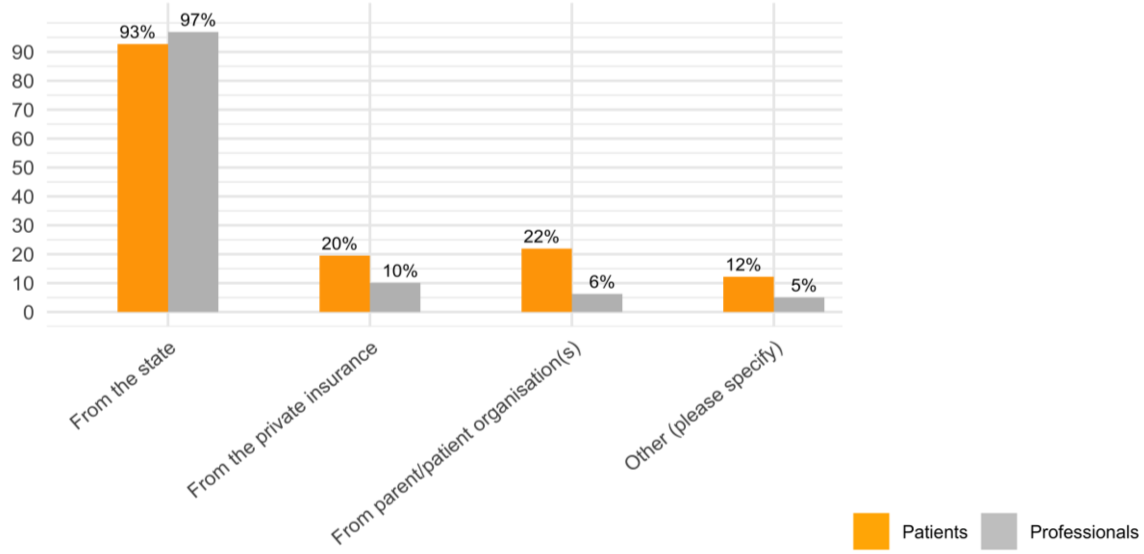

Are parents/patients/survivors informed about possible late effects in a timely and age-appropriate manner?

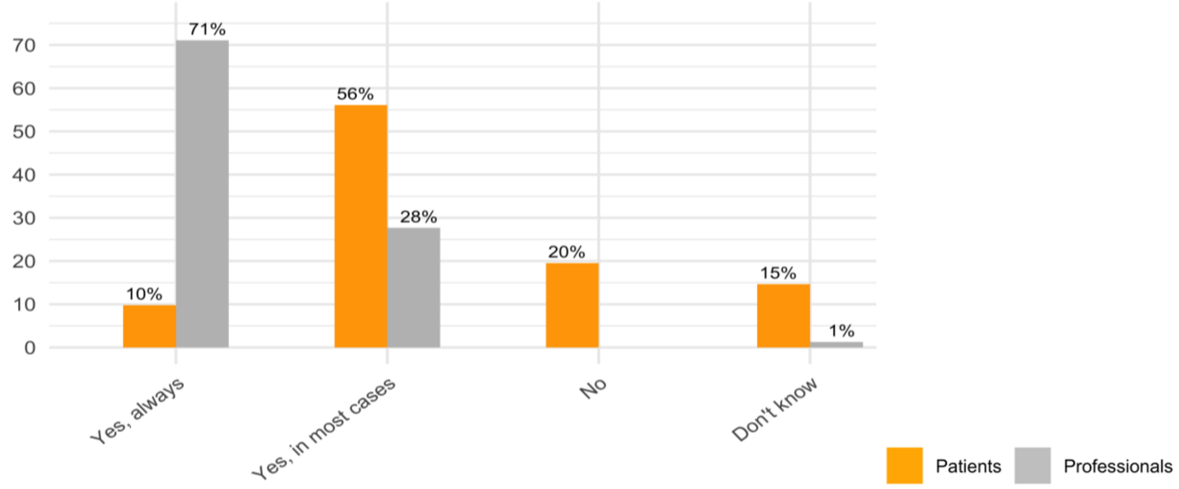

Are there any paediatric palliative care services available in your centre?

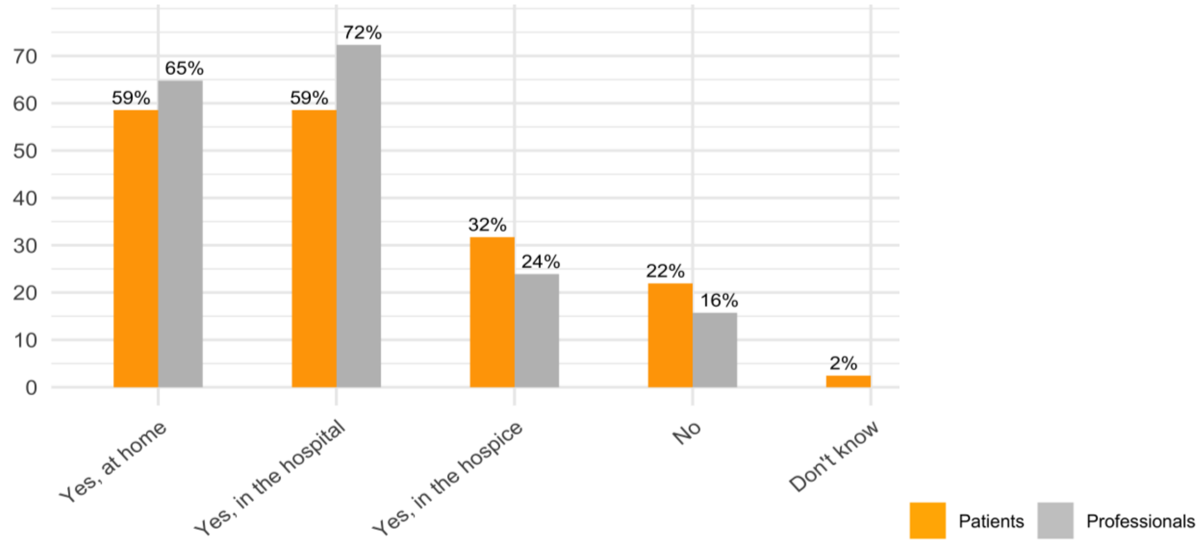

69: When is palliative care integrated into the care pathway in your centre?

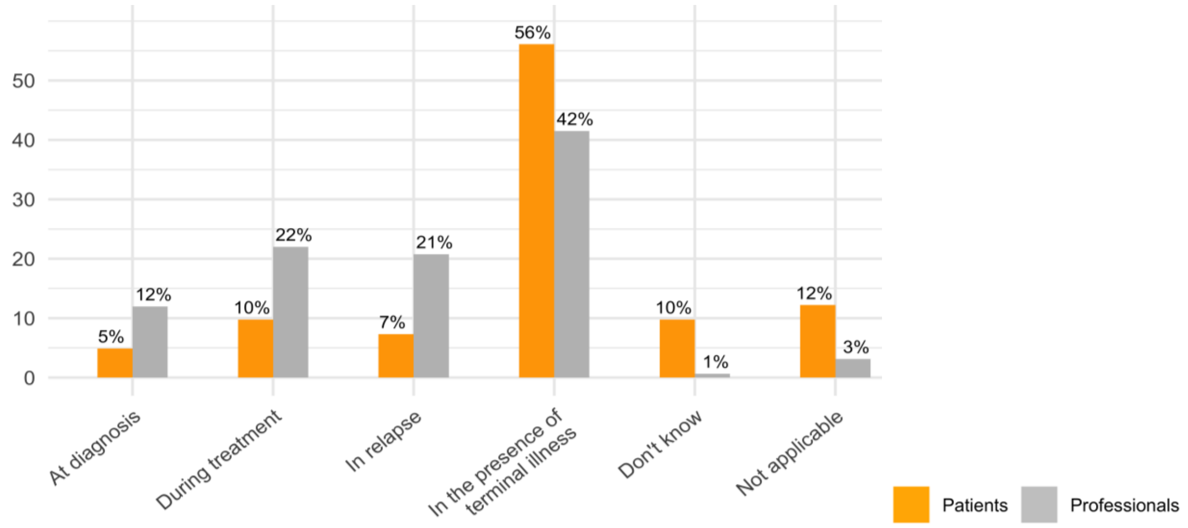

Supplemental S4: Two example answers stratified by country

Have you ever used the European Standards of Care for Children with Cancer?

Answer CCI-Europe:

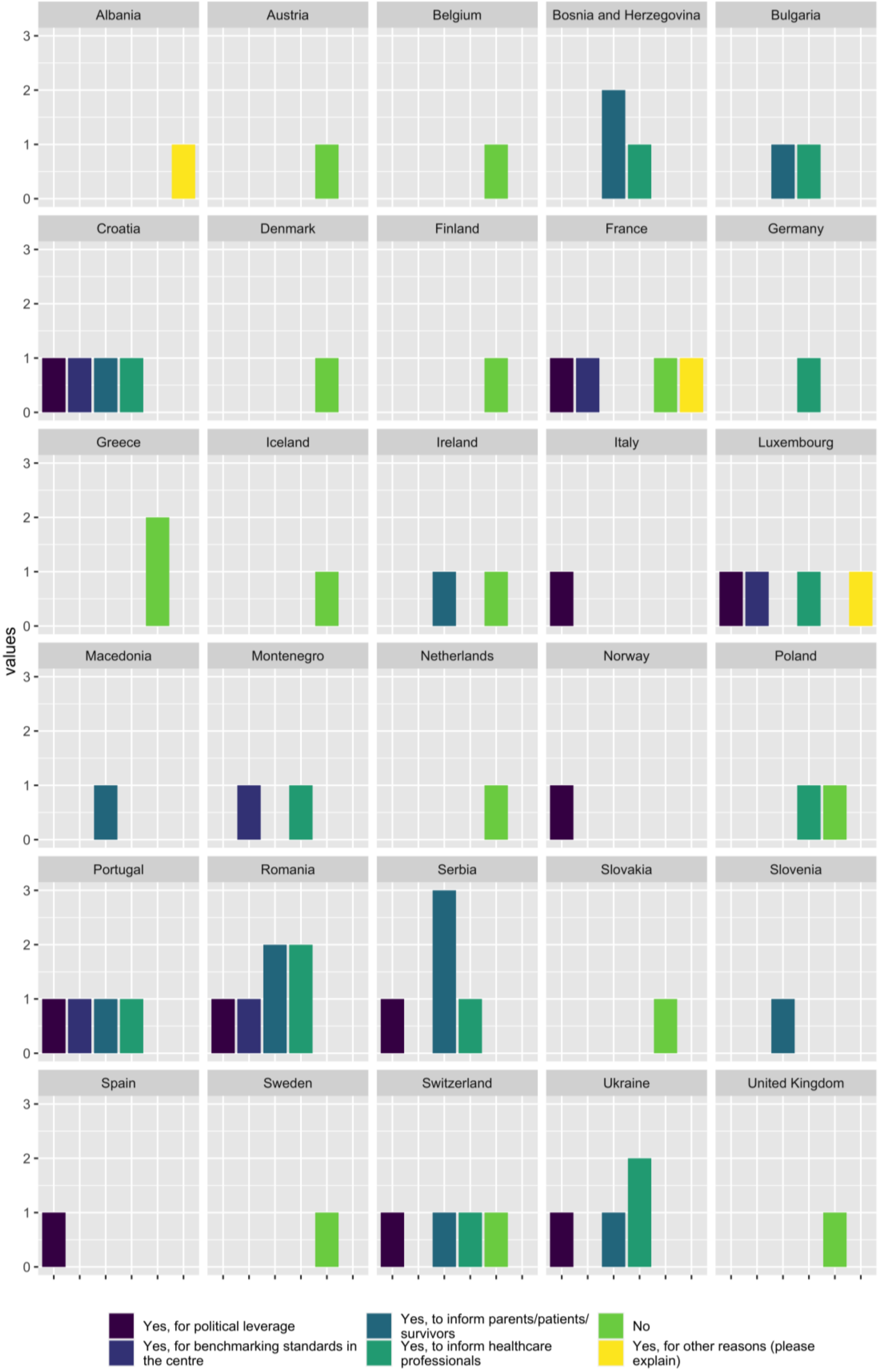

Answer SIOPE:

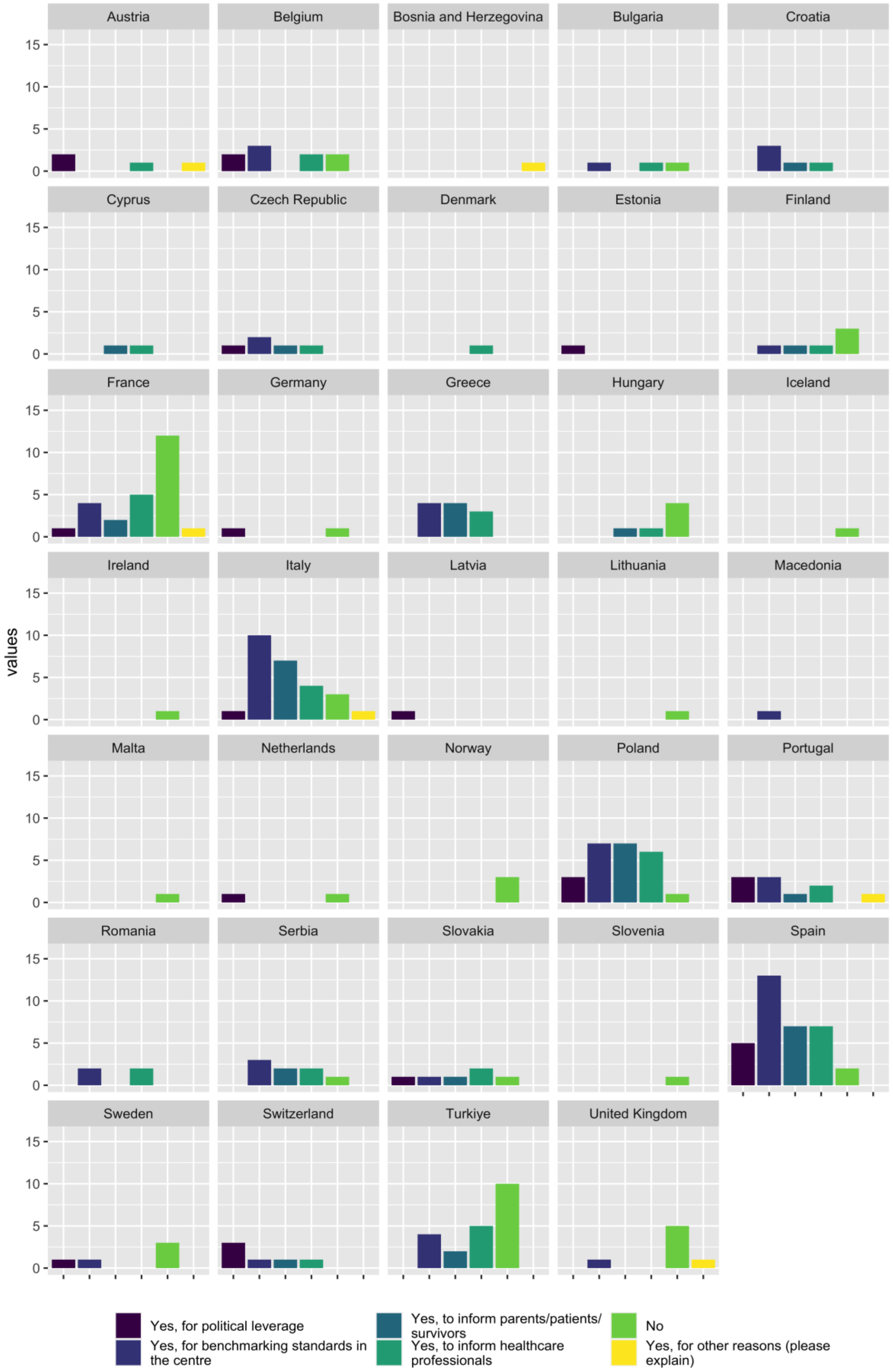

Is there a support system in place for patients and their families, e.g. to help them with social, administrative, financial, and legal issues, advise them when the first diagnosis is made?

Answer CCI-Europe

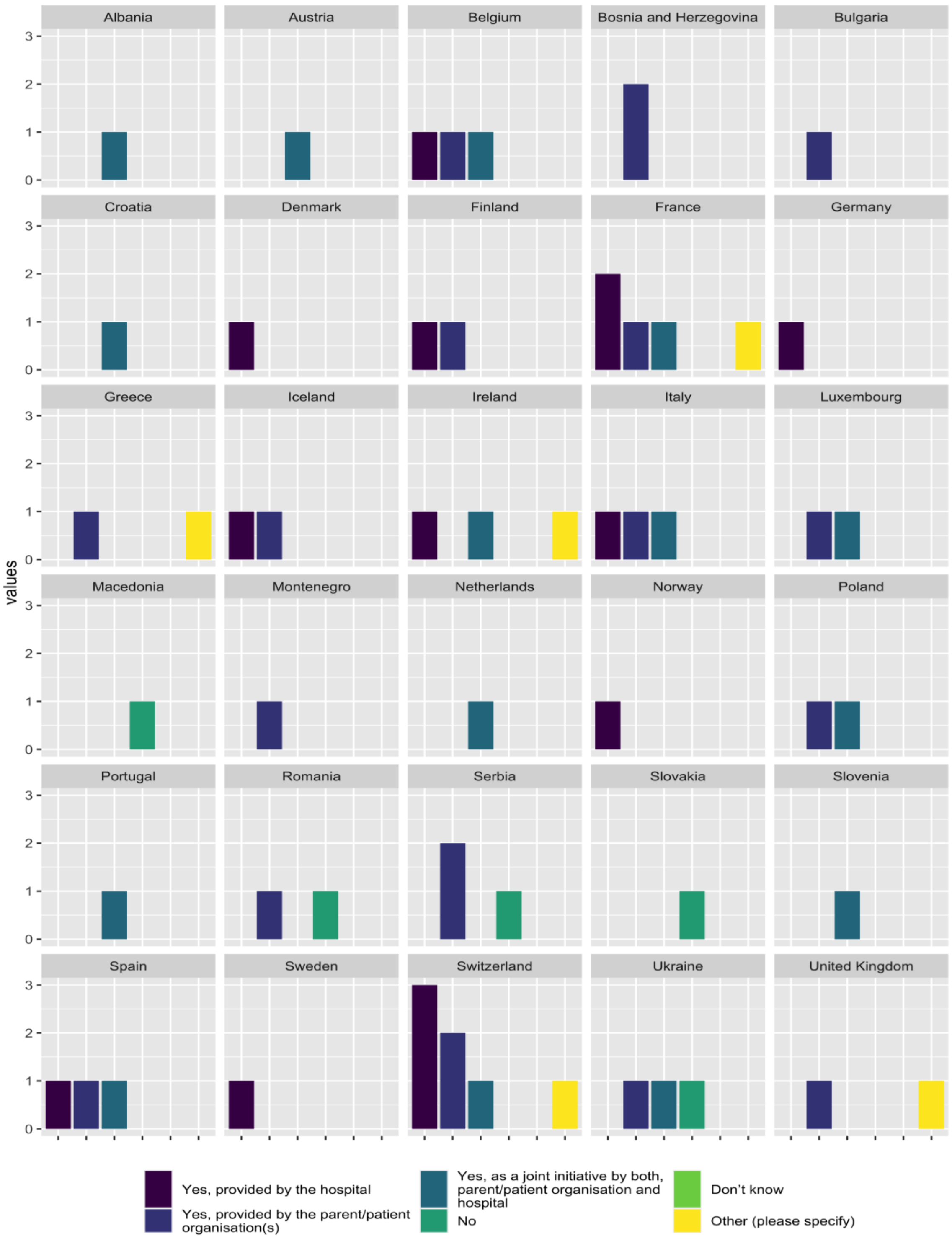

Answer SIOPE

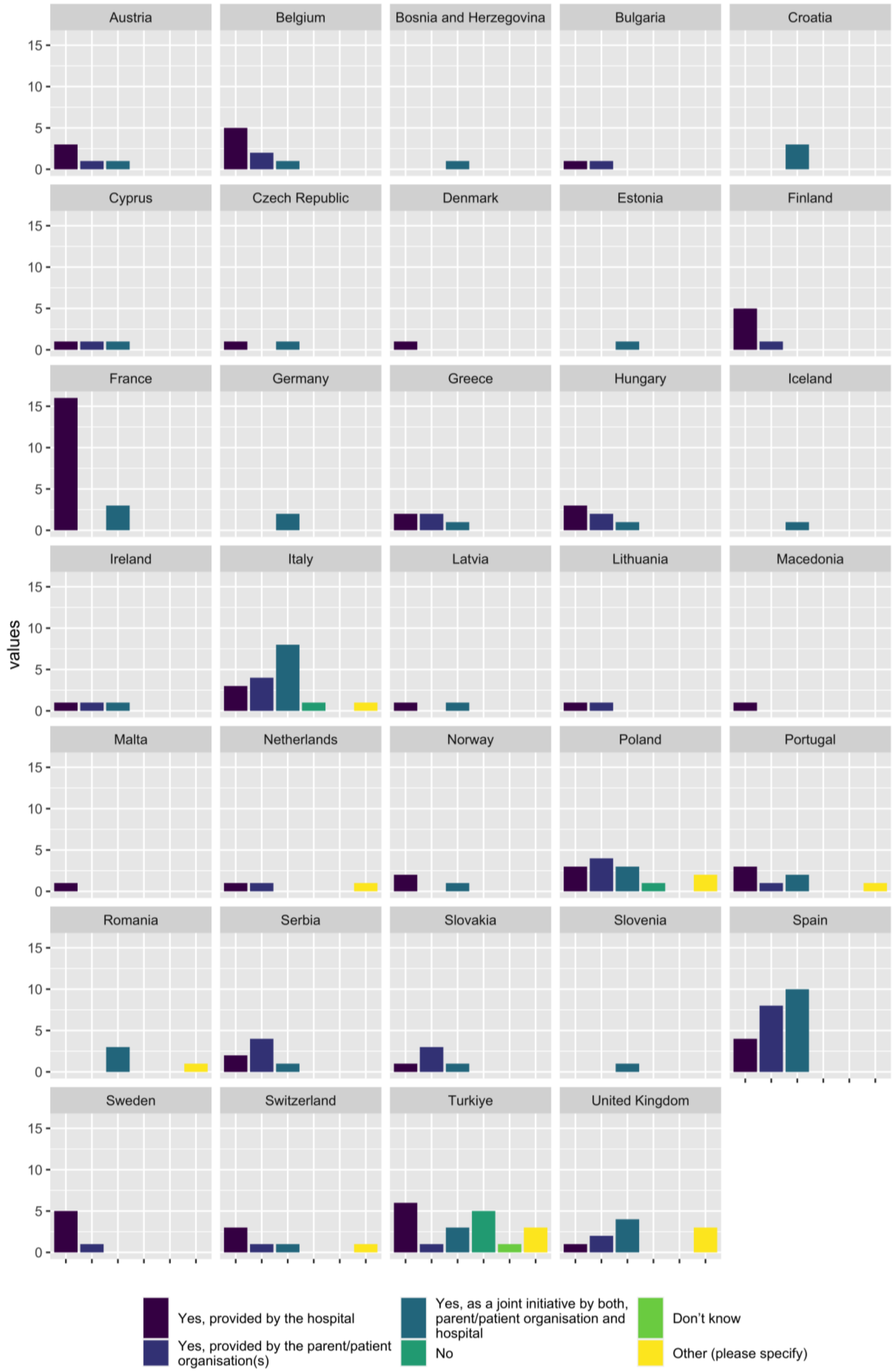

Supplement: Supplementary file 1 [file curroncol-32-00084-s001.zip › curroncol-3437099-supplementary.pdf]
